# Understanding the impact of distance and disadvantage on lung cancer care and outcomes: a study protocol

**DOI:** 10.1186/s12885-024-12705-9

**Published:** 2024-08-02

**Authors:** Daisy McInnerney, Samantha L. Quaife, Samuel Cooke, Lucy Mitchinson, Zara Pogson, William Ricketts, Adam Januszewski, Anna Lerner, Dawn Skinner, Sarah Civello, Ros Kane, Ava Harding-Bell, Lynn Calman, Peter Selby, Michael D. Peake, David Nelson

**Affiliations:** 1 Centre for Cancer Screening, Prevention, and Early Diagnosis, Wolfson Institute of Population Health, Queen Mary University of London, Charterhouse Square, London, EC1M 6BQ UK; 2 Lincoln Institute for Rural and Coastal Health, College of Health and Science, University of Lincoln, Lincoln, UK; 3 Lincoln County Hospital, United Lincolnshire Hospitals NHS Trust, Lincoln, UK; 4 Barts Health NHS Trust, London, UK; 5 Pilgrim Hospital, United Lincolnshire Hospitals NHS Trust, Boston, UK; 6 School of Health and Social Care, College of Health and Science, University of Lincoln, Lincoln, UK; 7 Swineshead Patient Participation Group, Swineshead Medical Group, Boston, UK; 8 Centre for Psychosocial Research in Cancer, School of Health Sciences, University of Southampton, Southampton, UK; 9 School of Medicine, University of Leeds, Leeds, UK; 10 Lincoln Medical School, College of Health and Science, University of Lincoln, Lincoln, UK; 11 Glenfield Hospital, University of Leicester, Leicester, UK; 12 Cancer Research UK, London, UK; 13 Macmillan Cancer Support, London, UK

**Keywords:** Lung cancer, Patient experience, Informal carer, Rural health, Urban health, Qualitative research, COM-B model, Oncology

## Abstract

**Background:**

Lung cancer is the third most common cancer in the UK and the leading cause of cancer mortality globally. NHS England guidance for optimum lung cancer care recommends management and treatment by a specialist team, with experts concentrated in one place, providing access to specialised diagnostic and treatment facilities. However, the complex and rapidly evolving diagnostic and treatment pathways for lung cancer, together with workforce limitations, make achieving this challenging. This place-based, behavioural science-informed qualitative study aims to explore how person-related characteristics interact with a person’s location relative to specialist services to impact their engagement with the optimal lung pathway, and to compare and contrast experiences in rural, coastal, and urban communities. This study also aims to generate translatable evidence to inform the evidence-based design of a patient engagement intervention to improve lung cancer patients’ and informal carers’ participation in and experience of the lung cancer care pathway.

**Methods:**

A qualitative cross-sectional interview study with people diagnosed with lung cancer < 6 months before recruitment (in receipt of surgery, radical radiotherapy, or living with advanced disease) and their informal carers. Participants will be recruited purposively from Barts Health NHS Trust and United Lincolnshire Hospitals NHS Trusts to ensure a diverse sample across urban and rural settings. Semi-structured interviews will explore factors affecting individuals’ capability, opportunity, and motivation to engage with their recommended diagnostic and treatment pathway. A framework approach, informed by the COM-B model, will be used to thematically analyse facilitators and barriers to patient engagement.

**Discussion:**

The study aligns with the current policy priority to ensure that people with cancer, no matter where they live, can access the best quality treatments and care. The evidence generated will be used to ensure that lung cancer services are developed to meet the needs of rural, coastal, and urban communities. The findings will inform the development of an intervention to support patient engagement with their recommended lung cancer pathway.

**Protocol registration:**

The study received NHS Research Ethics Committee (Ref: 23/SC/0255) and NHS Health Research Authority (IRAS ID 328531) approval on 04/08/2023. The study was prospectively registered on Open Science Framework (16/10/2023; https://osf.io/njq48).

**Supplementary Information:**

The online version contains supplementary material available at 10.1186/s12885-024-12705-9.

## Background

Globally, lung cancer is a leading cause of cancer mortality and premature death, particularly within communities experiencing deprivation, and is the third most common cancer in the UK [1, 2]. NHS England commissioning guidance for optimum lung cancer care recommends management and treatment by a specialist team, with a concentration of experts in one place, providing access to specialised diagnosis and treatment facilities [3]. However, the complex and rapidly evolving diagnostic and treatment pathways for lung cancer, along with workforce limitations, make achieving this goal challenging [4]. There are wide variations and inequalities in lung cancer care and survival outcomes across the UK [5]. Indeed, the deprivation gap (i.e. the survival difference between individuals from the least deprived compared to the most deprived groups) is highest for smoking related cancers, such as lung cancer, compared to other-cancer types [6]. Evidence suggests that, in the UK, poorer survival rates for people with lung cancer experiencing deprivation compared to more affluent groups are driven by lower screening and treatment rates [7, 8]. Broader, structural inequalities related to tobacco-dependence [9] also drive higher rates of lung cancer incidence and poorer outcomes for people experiencing socio-economic deprivation [10]. To close this deprivation gap in lung cancer, it is therefore vital to understand and address the factors underlying these lower treatment rates.

One integral factor to consider is patient engagement. Whilst the term ‘patient engagement’ is widely used and may have different meaning across different contexts [11], in this study, we define this term as the extent to which an individual patient attends, understands and undergoes each investigation, test and treatment that comprises their personal lung cancer pathway, as was recommended by, and agreed mutually with, their clinical care team. Enhancing and supporting patient engagement can improve patient outcomes and care experiences [11], and in the context of lung cancer may enable greater adherence to recommended diagnostic and treatment pathways. Most work to date has focused more so on improving lung cancer outcomes [12, 13] and the quality of the lung cancer services themselves [14], whilst neglecting to consider how the individual circumstances of people with lung cancer may impact their engagement with available services and support. For example, factors such as an individual’s location in relation to their lung cancer services; their available resources; their language and culture; their prior experiences of and beliefs about healthcare; and availability of social support, may all influence their engagement with care [15, 16].

Such factors can be explored systematically using behaviour change models, like the COM-B framework. This theoretical behaviour system model describes three essential and interacting conditions that determine how likely it is that an individual will perform a behaviour (B): their capability (C), opportunity (O) and motivation (M) [17]. Understanding these interacting factors in relation to individuals’ engagement with their recommended diagnostic and treatment pathway is crucial to identify how best lung cancer patients can be supported to take part in their recommended pathway. For example, one potentially helpful approach is Pathway Navigation. Cancer Alliances report that appointing Pathway Navigators, who provide tailored, individual support to help patients navigate and thus engage with their complex diagnostic test, appointment, and treatment schedules, can double the number of patients receiving lung cancer treatment within the target of 49 days [18]. Support with pathway navigation may be particularly crucial for individuals who do not have access to informal support from friend or family carers [19]. Equally, it is critical to understand the experiences of informal carers who are supporting people with lung cancer, to identify areas where additional support from formal healthcare services may be required.

The location of patients relative to the healthcare services they need to access is a particularly important element to consider in relation to an individual’s engagement in their diagnostic and treatment pathway. For instance, services situated in and serving rural or urban areas, are associated with both distinct and overlapping challenges to engagement. The UK consists of large rural and coastal populations that are often characterised by high levels of economic and social deprivation, limited digital infrastructure, poor mental and physical health, high smoking prevalence, and drug and alcohol misuse [20, 21]. The Chief Medical Officer for England has recently recognised the importance of better understanding the impact of place on health as well as the urgent need to address health inequalities in rural and coastal areas [22, 23]. The challenges faced by rural and coastal communities are often further exacerbated by poor access to healthcare (i.e. long travel distances, poor transport infrastructure, lack of available services) [24–27] and workforce limitations (i.e. poor recruitment and retention of healthcare professionals) [28, 29]. Urban areas of the UK also experience high levels of economic and social deprivation but typically in more concentrated areas characterised by diverse ethnic communities [30]. Urban communities also face significant mental and physical health challenges related to unique health inequalities including high population density and heterogeneity [31], elevated crimes rates [32], air pollution [33], lack of green spaces [34], and poor and unstable housing [35]. Whilst healthcare access, infrastructure and workforce are typically more developed in urban areas, highly specialised clinical teams are often situated in different hospital settings, requiring significant patient travel and time commitment. Although distances between centres are often shorter than in more rural settings, urban transport systems can be disparate, expensive, and complex to navigate.

There is a distinct lack of research surrounding lung cancer within UK and European settings compared to other tumour sites [36, 37]; especially qualitative inquiries that explore experiences across local settings. Further research is needed to gain an in-depth understanding of the individual-level barriers that urban, rural and coastal people living with and affected by lung cancer in the UK face, and to identify facilitators to support engagement. In this study, we will compare and contrast the challenges faced by people with lung cancer and the friends and family members who support them, in urban North East London, to those of predominantly rural and coastal Lincolnshire. The behavioural-science informed approach, theoretically underpinned by the COM-B model, will enable the identification of modifiable factors amendable to intervention to facilitate equitable engagement with the diagnostic and treatment pathway for lung cancer. The aims of this pragmatic and uniquely translational study are:

1. To explore how lung cancer patients and their informal carers (close family and friends who support people with lung cancer) characteristics and their location in relation to specialist services impact on their capability, opportunity and motivation to attend and participate in their recommended lung cancer diagnostic and treatment pathway in North East London and Lincolnshire.
2. To generate translatable evidence from both North East London and Lincolnshire to inform the evidence-based design of a patient engagement intervention to improve lung cancer patients’ and informal carers’ participation and experience of the lung cancer care pathway.

## Methods/Design

### Study design

This study will use a cross-sectional qualitative interview study design to explore the experiences of people with lung cancer and their informal carers in urban (North East London) and rural (Lincolnshire) areas of England. Guided by the Medical Research Council’s (MRC) framework for the development and evaluation of complex interventions [38], this study will be conducted in accordance with the intervention ‘development’ phase of the framework, by generating translatable evidence to inform the evidence-based design of a patient engagement intervention that will aim to better support lung cancer patients in engaging in treatment and care pathways. In this study, we are defining patient engagement as the extent to which an individual patient attends, understands and undergoes each investigation, test and treatment that comprises their personal lung cancer pathway, as was recommended by, and agreed mutually with, their clinical care team. This study will be reported in line with the Consolidated Criteria for Reporting Qualitative Research (COREQ) checklist [39].

### Study setting

The study will be conducted in an urban (North East London) and rural and coastal area (Lincolnshire) of England, United Kingdom. It should be noted that the county of Lincolnshire also has urban areas such as the city of Lincoln, although the county as a whole, is predominantly rural in geography, with a significant coastline to the East. People diagnosed with lung cancer and their informal carer’s will be recruited from two NHS trusts: Barts Health NHS Trust and United Lincolnshire Hospitals NHS Trust. Barts Health NHS Trust consists of five hospitals in the City of London and East London (Mile End Hospital, Newham University Hospital, Royal London Hospital, St Bartholomew’s Hospital and Whipps Cross University Hospital) and serves a population of ~ 2.6 million people within an urban area. United Lincolnshire Hospitals NHS Trust consists of four hospitals that cover the county of Lincolnshire (Lincoln County Hospital, Grantham and District Hospital, Pilgrim Hospital Boston, and County Hospital Louth) and serves a population of ~ 700,000 people across a predominately rural area. In the case of Lincolnshire, some people with lung cancer are referred to Nottingham City Hospital as part of Nottingham University Hospitals NHS Trust (NUH) for treatment. Nottingham City Hospital is located in the city of Nottingham within the East Midlands region of England and is located approximately 43 miles from Lincoln city and 80 miles from the East coast of Lincolnshire. Poor road conditions and a lack of accessible public transport can make traveling from the more rural and coastal parts of Lincolnshire to Nottingham, both costly and time consuming [40]. NUH staff from Nottingham City Hospital will support the identification and recruitment of people with lung cancer and their informal carers who have been referred from United Lincolnshire Hospitals NHS Trust sites for treatment.

### Ethical approval and study registration

The protocol for this study was registered on Open Science Framework on October 16th, 2023 (https://osf.io/njq48). Ethical approval was obtained (REC Ref: 23/SC/0255; IRAS ID:328531) from the NHS Oxford B Research Ethics Committee and the NHS Health Research Authority on August 4th, 2023.

### Theoretical approach

An exploratory qualitative approach underpinned by the COM-B Model for Behaviour Change [17] will be applied. This will enable the identification of factors potentially amendable to intervention to initiate health behaviour change (i.e., to facilitate improved engagement with the recommended lung cancer diagnostic and treatment pathway). A person-centred pragmatic epistemological approach will be taken [41], unpinned by the view that knowledge is based on experience, whilst recognising the unique knowledge of each individual as created by their unique experiences. The pragmatist epistemology supports combining inductive and deductive approaches, and selection of research methods based on their appropriateness for addressing real-world problems [41, 42]. Here, qualitative interviews and combined inductive and deductive framework analysis have been selected. This will enable inductive analysis of individual’s unique experiences, challenges and needs; mapped deductively to domains of behaviour change, to generate in-depth, person-centred, translational insights. These in turn will be applied to inform development of a pragmatic intervention to address existing inequities in engagement with the recommended lung cancer pathway. By prioritising a ‘practical understanding’ of these issues, this approach will allow us to understand and address the unique challenges and practical needs of people with lung cancer and their informal carers in urban, rural, and coastal areas.

### Participants

This study will recruit people with a confirmed diagnosis of lung cancer within the last six months from three patient cohorts who are in receipt of (1) surgery (2) radical radiotherapy or (3) currently with advanced disease, including both those having active anticancer treatment and specialist palliative/best supportive care (provided they were eligible for treatment). The criterion of six months was chosen because the insights will inform a patient engagement intervention to be delivered early in the diagnostic and treatment pathway (i.e. during or close to the first lung cancer clinic appointment). It is therefore important that participants can recall their experiences of the earlier phases of the investigation and treatment pathway, whilst balancing this with their treatment burden and ability to participate. This study will also recruit informal carers of people with lung cancer with a confirmed diagnosis within the last six months. People diagnosed outside of this timeframe, who do not have capacity to provide informed consent or who are not able to understand the recruitment materials (i.e., participant information sheet/video and informed consent form) with assistance of an interpreter are not eligible to participate in this study.

### Sampling

This study will aim to recruit up to 60 patients and 30–60 informal carers (at least 15 at each site) across both NHS trusts, split evenly between United Lincolnshire Hospitals NHS Trust and Barts Health NHS Trust. We will use a purposive sampling approach to achieve representation from patients receiving different types of treatment (surgical, radical radiotherapy, or advanced cancer) and informal carers. Once ten people have been recruited, recruitment will be targeted following a maximum variation purposive sampling framework [43] to ensure diversity within the sample in relation to: gender, ethnicity, age, socioeconomic position, stage of disease at diagnosis and area of North East London or Lincolnshire; and for informal carers, these factors along with the type of caring relationship they have to the patient (e.g., friend, family member). This type of recruitment will allow us to explore the experiences of a diverse set of participants and ensure the findings and recommendations are applicable to the diverse range of individuals who may be referred on a lung cancer pathway. The chosen sample size is in line with norms for qualitative research [44, 45]. This sample size is required due to the multi-site nature of the study and the diversity of the population [46–48]. The sample size is sufficient to achieve appropriate information power for a study which is well-designed, theoretically-grounded, and addressing specific objectives [48].

### Recruitment

Participant recruitment and data collection will run between November 2023 and May 2024. All participants will give their informed consent (i.e. either written or verbal) to take part prior to the start of each interview. At both sites, participants will give consent to a member of the research team who is experienced in qualitative interview methods.

#### People with lung cancer

Patient lists will be pre-screened for people who meet the eligibility criteria by a member of the direct care team at routine clinic meetings at both NHS sites. For each person who is eligible for the study, a member of their direct care team will give a brief overview of the study during their appointment and ask for their consent for a member of the research team to contact them directly. They will then be given and/or sent an information pack (an invitation letter, information sheet and reply slip) and invited to express an interest in taking part either by post, email, or telephone. The information pack will also contain a link to a video-version of the invitation letter, information sheet and reply slip that can be accessed online. In the case of North East London, the information pack, video-version, invitation letter, information sheet and reply slip will also be made available in Sylheti owing to the large Bangladeshi population living in the geographic area served by Barts Health NHS Trust. If the clinical team do not introduce the study to eligible participants during their appointment, they will receive an invitation and information pack by post. A note will be made on the clinical record once a person has received an invitation to ensure they are not re-invited, and to confirm whether they take part in the study.

#### Informal carers

The information packs distributed to eligible patients will also include information for informal carers, explaining that they are also invited to take part in a separate interview. The reply slip will include an option for either the patient, informal carer, or both to take part in the interview. The option for informal carers to take part will also be mentioned by health care professionals when they introduce the study during appointments and will be mentioned by a member of the research team on the phone to potential patient participants. Patients whose informal carers’ do not want to or are not able to take part in an interview themselves can still be recruited to the study, as can informal carers whose patients do not want to or are not able to take part in an interview themselves.

### Data collection

Interviews will be carried out by researchers (SC and LM) experienced in conducting qualitative research and audio-recorded on an encrypted recorder. Each interview will last approximately 1-hour and will take place face-to-face or via telephone or Microsoft Teams, depending on participant preference. We intend to only interview participants once, however, if they are tired or not feeling well during the interview, a follow up meeting can be arranged to complete the interview. Where possible, interviews with people with lung cancer and informal carers will be conducted separately to minimise social desirability bias [49]. However, as the interview explores sensitive subjects during an emotionally and physically challenging time of the person with lung cancer and informal carers’ lives (following a recent lung cancer diagnosis), the participant can request their patient/informal carer is present during the interview. In this case, the option to conduct the interview as a dyadic interview will be offered [50]. For participants who are not able to communicate clearly in English, an interpreter will be arranged to assist with both the phone calls to arrange the interview and the interviews themselves.

Interviews will follow a semi-structured topic guide (Additional file 1) developed by the research team, wider steering group and with patient and public involvement. The interviews will explore people with lung cancer and informal carers’ capability (physical and psychological), opportunity (physical and social) and motivation (reflexive and automatic) to participate in the lung cancer pathway, based on their individual characteristics and location in relation to the specialist lung cancer centres. This will include exploring factors associated with navigation of complex travel systems across multiple sites to attend appointments; attending and engaging with key touch-points along the pathway (including diagnostic processes; referral; systemic, radio-therapeutic and surgical treatments; palliative and allied health services); and digital consultations. The questions will be adapted for patient, informal carer, or dyadic interviews.

Demographic information including age, gender, ethnicity, post code (as proxy for region, rural-urban residence and socioeconomic position via Index of Multiple Deprivation score), stage of disease, and performance status will be extracted from the medical records of consenting participants. Additional questions in relation to participant characteristics and health behaviours (e.g. lifestyle, smoking behaviour) will be asked as part of the pre-determined interview schedule. The sample will be described in terms of; age, gender, ethnicity, disease stage, location (e.g. rurality/urban), area-level deprivation (converted from postcode to Index of Multiple Deprivation quintile), and performance status.

### Data analysis

Following completion of the interviews, the audio-recordings will be professionally transcribed, and a subset checked for accuracy. Transcripts will be psesudonymised and stored securely on the University of Lincoln’s One Drive and Queen Mary University of London’s Data Safe Haven. The qualitative data analysis software package NVivo will be used to support the analysis. A framework approach to applied thematic analysis, as described by Ritchie and Spencer (1994) [51], will be used to analyse qualitative data. Framework analysis is well-suited to analytical approaches involving multi-disciplinary team members and will enable comparison and interpretation of patterns of themes both within and between North East London and Lincolnshire. This approach will allow us to identify similarities and differences between the two sites, comparing factors affecting patient engagement in rural and urban settings. It will also enable systematic identification of potentially modifiable factors related to participants’ capability, opportunity and motivation to engage that can be targeted by a patient engagement intervention, as well as interactions between these factors and implementation considerations.

The framework method is a five-stage qualitative analysis process involving; (1) Familiarisation, (2) Identifying a thematic framework, (3) Indexing, (4) Charting, and (5) Mapping and Interpretation [51]. The coding of data will be guided by an inductive and deductive approach, allowing for a data-driven and theory informed development of an analytical framework. The analytical framework will be developed collaboratively between the North East London and Lincolnshire research teams, in consultation with the broader steering group and Patient and Public Involvement and Engagement (PPIE) representatives. The COM-B model will be used to guide the structure of the analytical framework, enabling the grouping of facilitators and barriers to participants’ engagement (capability, opportunity and motivation to engage) with the lung cancer pathway. The analysis will result in a set of recommendations for the proposed patient engagement tool, drawn from the analysis of both the Lincolnshire and North East London interviews, addressing both (a) common principles; and (b) region-specific recommendations. The quantitative demographic data will be summarised and presented as ranges and percentages to describe the overall sample.

### Study management and oversight

The study conduct is overseen by a national steering committee, who meet bimonthly to monitor progress, ensure alignment between research sites and with overall project aims, and contribute to results interpretation; application to intervention development; and dissemination. The steering committee is made up of funding body representatives from Cancer Research UK; researchers with expertise in qualitative methods, behavioural science and health inequalities from both Lincolnshire and London; clinicians (oncologists, respiratory physicians, and nurses); NHS cancer pathway managers and administrators; and PPIE representatives. The steering committee are also responsible for delivering a parallel quantitative service evaluation project, that the results of this qualitative study will inform. Alongside the national steering committee, two regional study management groups have been established to manage operational processes at both sites and inform data interpretation and PPIE collaboration.

### Reflexivity

Qualitative research is contextual and we as a diverse team of clinical and non-clinical researchers, healthcare professionals and people with lived experience, recognise the importance of reflexivity as a crucial strategy in the process of generating knowledge via qualitative research [52, 53]. Reflexivity is considered a major foci for quality control and understanding how it may influence a study should be carefully considered [52]. Where researchers clearly describe the contextual intersectional relationships between the participants and themselves, this can improve the robustness of the study and generate a deeper understanding of the findings [53]. This study takes place within two distinct geographic settings, the predominantly rural and coastal county of Lincolnshire and urban North East London. The context is the delivery of lung cancer care in both these settings and the experiences of people diagnosed with lung cancer care and their informal carers who reside in both Lincolnshire and North East London. Both areas have unique social and environmental contexts but are linked by inequalities in lung cancer care. North East London is the London region with the highest level of deprivation and an ethnically diverse community, with over two-thirds of the community from a minority ethnic group. These factors are associated with higher lung cancer mortality and challenges navigating complex healthcare pathways [5, 6]. Lincolnshire is not as ethnically diverse with the majority of the population being White British although there is a sizeable Central and Eastern European community. Access to lung cancer care or oncology care for people living in rural and coastal areas is hindered by the uneven geographic distribution of workforce and services [37, 54]. We therefore have site-specific research teams that possess a wealth of subject-specific and methodological expertise as well as individual and collective experiences of residing and/or working in these two sites. Reflexivity will also be carefully considered throughout the study by maintaining reflexive logs to document evolving thoughts, biases, and personal reflections during data collection. These will be shared with the wider team at regular team meetings to promote wider reflexivity insights and will be used to help frame and contextualise the interpretation of and meaning of data. The teams will meet regularly throughout data analysis and interpretation, with meetings minuted and reflected upon, to inform the iterative analysis approach and provide core contextual reflections that will be reported with study findings.

### Patient and public involvement and Engagement (PPIE)

This study was developed in response to a need identified by Cancer Research UK based on foundational PPIE focus groups, and PPIE is embedded throughout the study lifecycle. The protocol was developed and co-authored by a public contributor with lived experience as a lung cancer carer (AH-B). The study documents (invite, information sheet, consent form and draft interview questions) have also been reviewed by two people living with lung cancer and one carer using a PPIE consultation sheet (Additional file 2) and facilitated by our NHS colleagues. Two region-specific PPIE groups have been established; members have lived experience of living with lung cancer, or as informal carers providing support to individuals living with lung cancer. These groups will be consulted at key points throughout the study lifecycle and will play a crucial role during the conduct of this research through providing unique perspectives, support, and guidance. We will also work with these groups to report our findings in accessible formats informed by patients and informal carers’ needs. There will also be opportunities for patients and informal carers to support the dissemination of our findings to clinical and non-clinical audiences. Where appropriate, there will be opportunities for interested members to collaborate and co-author both academic and non-academic outputs. The involvement of PPIE members will extend beyond the conclusion of this study and will play an integral role is shaping and refining the patient engagement tool throughout its subsequent development phases.

### Dissemination

We will publish the study findings in peer-reviewed scientific journals and present them at appropriate national and international conferences. Accessible summaries will also be produced and disseminated to people with lung cancer, informal carers, and healthcare professionals. A detailed dissemination plan for this study has been created and agreed upon by the study steering committee. PPIE representatives contributed to the dissemination plan development to ensure our findings will be shared in an inclusive and community-focused way.

## Discussion

This qualitative cross-sectional study will address an urgent need to better understand the experiences and difficulties of lung cancer patients and their informal carers who reside in urban, rural and coastal areas of the UK. More specifically, this study will gather important insight into the capability, opportunity, and motivational factors that may influence lung cancer patients’ engagement in optimal care pathways in these settings. Recent systematic mapping of global cancer screening, prevention, and diagnosis research between 2007 and 2020 points towards a clear disparity in the volume of cancer research across tumour site, with 61% of included studies (*n* = 1762) conducted in colorectal, breast and cervical cancer [36]. Despite being the leading cause of cancer related deaths globally [2], only 6.4% percent of studies were in relation to lung cancer [36]. Furthermore, evidence suggests that our understanding surrounding the development of and engagement in optimal care pathways for people with lung cancer remains in its infancy across the broader health systems [4], highlighting the need to better understand how individuals engage in these pathways across local settings [4]. Whilst the world’s largest independent cancer research organisation, Cancer Research UK have prioritised lung cancer research over the last decade [55], our understanding of lung cancer within a UK context predominantly stems from epidemiological and quantitative inquiries. There remains a dearth of evidence that explores the qualitative experiences of people living with lung cancer who reside in both rural and urban areas in the UK. This is particularly evident in rural settings with recent review evidence identifying only a limited number of qualitive studies (*n* = 9 studies) undertaken in rural areas none of which were from a UK or European setting [56].

Cancer care pathways are becoming increasingly challenging to deliver and engage with due to their rapidly evolving and complex nature [57], as well as the multi-faceted individual-level barriers faced by patients unique to urban and rural settings. By identifying and understanding these factors, the study findings can inform the development of tailored services to enable more personalised and patient-centred lung cancer care. Indeed, evidence generated by this study will directly inform the development of a patient engagement intervention that will aim to support lung cancer patients to optimally engage with their recommended care pathway. The MRC has published, and recently updated, their guidance surrounding the development and evaluation of complex interventions, presenting a framework of four phases: (1) development, (2) feasibility/piloting, (3) evaluation, and (4) implementation [38, 58]. The current study forms an integral element as part of the ‘development’ phase of the MRC framework, with the qualitative interview findings iteratively integrated with insights from a series of region-specific key stakeholder workshops with a range of healthcare professionals; service managers and co-ordinators; and PPIE representatives. This approach will ensure that experiences and perceptions are gathered from stakeholders across the care continuum to inform robust, patient-centred and theory-and-evidence-based intervention development, with core implementation factors considered throughout. Once we have developed the key components of the patient engagement intervention, we plan to then undertake iterative feasibility and acceptability testing in late 2024 / early 2025. This will be followed by intervention evaluation where we will ascertain the impact of the intervention on key quantitative indicators of pathway engagement, as well as qualitative exploration of patient and carer experience.

### Electronic supplementary material

Below is the link to the electronic supplementary material.

Supplementary Material 1

Supplementary Material 2

## Data Availability

The dataset(s) that will support the conclusions of this article will be included within the article and its additional file(s).

## Abbreviations

COM-B: Capability Opportunity Motivation Behaviour Model
GDPR: General Data Protection Regulation
NHS: National Health Service
PPIE: Patient and Public Involvement and Engagement
UK: United Kingdom
ULHT: United Lincolnshire Hospitals NHS Trust
