# Supplementary material for: Understanding the impact of distance and disadvantage on lung cancer care and outcomes: a study protocol
Source: BMC Cancer. 2024 Aug 2;24:942. doi: 10.1186/s12885-024-12705-9 (PMC11295610; doi:10.1186/s12885-024-12705-9)
Supplement: Supplementary file 1 — Supplementary Material 1 [file 12885_2024_12705_MOESM1_ESM.docx]

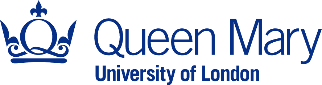

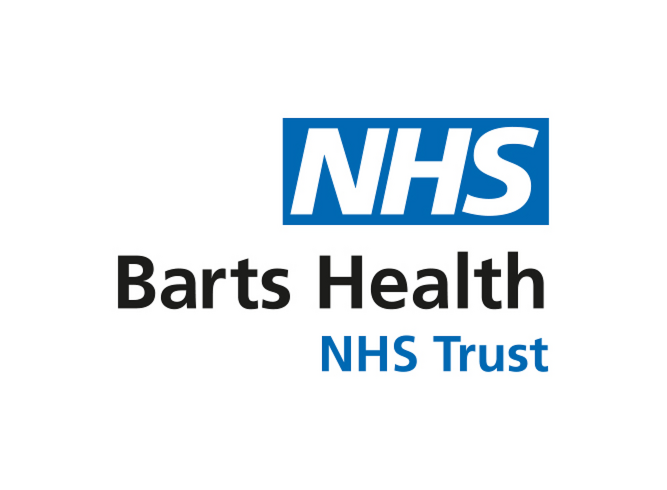

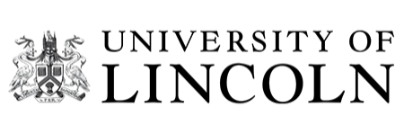

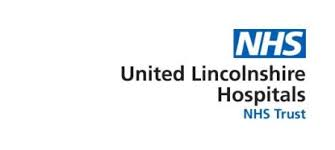


**Seeking patient and public views about a research study exploring how people access lung cancer services in urban and rural areas**

We are working with Cancer Research UK and researchers from Queen Mary University of London and University of Lincoln on an important study. The study looks at how personal disadvantage and distance from the location of specialist services impact on lung cancer care and patient outcomes.

We are seeking feedback on some draft materials that we plan to use in the study.

**About the study:**

We will be interviewing people living with lung cancer (and people who support them, e.g close family and friends) to ask them about their experiences accessing lung cancer care. We will be interviewing people in North East London and in Lincolnshire, to compare experiences in urban and rural settings. These will be one-off interviews conducted in-person, online or over the phone. The findings from the study will be used to develop a tool to help people with lung cancer more easily access their lung cancer care.

**Why we are asking for your help:**

It is critical to have Patient and Public Involvement (PPI) embedded in research projects from the outset to help design the study. Public involvement representatives can provide insights into what it is like to live with or care for someone with a particular illness. These insights can help to make health research more relevant to the needs of patients.

**What are we asking of you?**

1. **Please read the following materials. Please note that these are early drafts that we will update following feedback from patients and the public:**

- Invitation letter
- Participant Information Sheet
- Consent form
- Ideas for interview questions

1. **Please let us know what you think about them by answering the 5 questions on the next pages of this document.**

You can feedback in whatever way is easiest for you – ideally by **Wednesday 26^th^ April**. You could:

- type your answers directly into this document in the space provided and email back to the University of Lincoln team **OR**
- leave comments using Track Changes directly on the draft materials **OR**
- email your feedback directly to the University of Lincoln team **OR**
- arrange to speak to us by phone to feedback

**University of Lincoln research team contact details**

Email David Nelson from the University of Lincoln research team: dnelson@lincoln.ac.uk

Phone David from the University of Lincoln research team: 01522 837343

**Questions about the study materials**

| 1. **What are your first thoughts about this research?**   Please share any of your general thoughts – should it be done? Is it worthwhile or not? |
| --- |
| Please type here |
| 1. **What are your thoughts about the invitation letter:**   ***How clear is it? Are there any parts that do not make sense? What parts would you change and why?***  The invitation letter will be sent to all potential participants who express an interest in the research following an introductory conversation with their lung cancer care team. The letter will be sent out along with the information sheet and consent form. |
| Please type here |
| 1. **What are your thoughts about the Participant Information Sheet:**   ***How clear is it? Are there any parts that do not make sense? What parts would you change and why?***  Researchers will go through this information with potential research participants before they sign up to take part in the study. This is so the potential participant has a clear idea of what taking part in the study involves, as well as the risks and benefits. This enables the participant to make an informed choice about whether or not they choose to take part. |
| Please type here |
| 1. **What are your thoughts about the consent form?**   ***How clear is it? Are there any parts that do not make sense? What parts would you change and why?***  Researchers will go through this form with participants to ensure there is a record that all people taking part in the research have understood the participation information sheet and are clear about:  - What is being asked of them   - How information about them collected about them in the study will be used |
| Please type here |
| 1. **What are your thoughts about the ideas for interview questions?**   ***How clear are the questions?***  ***Do you think these questions would be acceptable to ask a person living with lung cancer as part of this study?***  ***Are there any more questions you think would be helpful to ask? Why?***  ***Are there any questions you don’t think we should ask? Why?***  ***Is there anything else you would change about the topic guide?***  The researcher will use the questions in the topic guide to guide the interview with the research participant. This helps the researcher make sure that the interview gathers information from the participant, in a way that:   - Meets the aims of the study AND - Is acceptable for the participant |
| Please type here |

**About you**

| We are collecting this information so that we can understand the characteristics of those who have kindly shared their views with us. | |
| --- | --- |
| **What age are you?** | Less than 40 years  40 – 60 years  60 - 80 years  Over 80 years  Prefer not to say |
| **What gender are you?** | Male  Female  Other gender (use this space to self-describe if you would like to share)______________  Prefer not to say |
| **Do you have or have you ever had cancer?** | Yes, I have/have had lung cancer  Yes, I have/had a different type of cancer  No  Prefer not to say |
| **Do you have or have you ever helped care for a friend or family member with cancer?** | Yes, I care for/have cared for somebody with lung cancer  Yes, I care for/have cared for somebody with a different type of cancer  No  Prefer not to say |
| **Would you be willing to help feedback on other materials as part of the design of this study?** | Yes  Maybe  No |
