# Supplementary material for: Understanding the impact of distance and disadvantage on lung cancer care and outcomes: a study protocol
Source: BMC Cancer. 2024 Aug 2;24:942. doi: 10.1186/s12885-024-12705-9 (PMC11295610; doi:10.1186/s12885-024-12705-9)
Supplement: Supplementary file 2 — Supplementary Material 2 [file 12885_2024_12705_MOESM2_ESM.docx]

**Patient Interview Topic Guide**

**Introductory questions**

Please could you introduce yourself.

Tell me about the area where you live.

**Questions on/experience of referral route**

When did you first develop/notice symptoms?

Did you have respiratory symptoms?

When and how were you diagnosed? What was your confirmed diagnosis?

Did your GP or healthcare professional discuss the possibility of you having lung cancer?

What was your wait time from your diagnosis to seeing an oncologist?

When were you referred for treatment following your diagnosis?

Were you informed that you may need to make multiple journeys for investigations?

Were you informed of the timescales for reporting investigations?

What types of test were involved in the diagnosis process? X-ray, CT scan, PET-CT scan, Biopsy?

How easy was it to get an appointment, how long did this take? Did the timescales seem appropriate/reasonable/realistic?

Experiences on the conversation/communication around diagnosis/treatment with primary care/GP/healthcare professional and specialist?

**Knowledge and willingness to adhere to necessary elements of a diagnostic and treatment pathway**

What options were made available to you for your treatment?

- Chemotherapy, Radiotherapy, Surgery, Targeted Therapy, Immunotherapy

How much did you understand about these options? Were you informed that you may need to travel for some treatments?

What aspects of treatment were explained to you? (prompts: side effects, effectiveness)

How involved did you feel in the discussions about your options and the final treatment decision? How involved did you want to be?

What did you want to do with regards to your treatment? Did this differ from your treatment plan? How did you make a decision about your treatment?

- Time/frequency of treatment
- Perceptions of treatment efficacy
- Possible side effects and tolerability
- Effects of treatment on family/social life
- Impact on work/finances/caring responsibilities
- Health status at the time (e.g., comorbidities, lung cancer symptoms)
- Opinion/recommendation of clinician
- Experiences of others/existing perceptions of treatment
- Views/influence of family/friends.

Who did you speak to/consult about your diagnosis/treatment?

Can you talk me through your experience of those conversations?

Were you aware of any support? What types? Did you want to/were you able to access this support? Macmillan Cancer Support? What support do you have at home?

Did you meet one of the Macmillan team pre/post diagnosis and offered support? Was this of benefit?

Who was your go to person to support you with the anxiety or worries or going to see a specialist oncologist?

If you went out of county/area for support or a specialist clinic, what support did you received from Lincolnshire/NHS Trust?

Can you think of anything that could have been done better about your diagnosis/treatment or that was done well and should be continued?

Can you talk me through your experience of travelling to and from your treatment appointments?

- Ease/difficulty
- Talk me through how you made your journeys/experiences of them
- How far did you (would you have been prepared) to travel for your treatment?
- How did you feel about that?
- Did you have/want someone to go with you?
- Do you have access to a vehicle/public transport?
- Financial impact of treatment? (i.e., travel costs, lost work/other implications)
- Impact of health status/wellness/symptoms/other long-term or health conditions.
- Views on time commitment
- Impact of competing responsibilities/commitments
- Managing different appointments at different places with different teams?
- Consistency and clarity (or not) of correspondence about appointments

What impact did your diagnosis and treatment have on your family/partner/children/friends?

You decided not to do [X], could you tell me about the reasons behind that decision? Is there anything that could have been done differently?

Decision making – how location has influenced this?

Health status – wellness/ability to travel

COM-B model – motivation

Perceptions of treatment efficacy and tolerability

Other people’s experiences – perhaps using third person to frame questions.

Towards end – suggestions, thinking about patients in the future, anything living in your area you would want?

Smoking behaviour – Health professionals’ attitudes towards smoking?

‘Myths’

Using data/evidence to frame questions ‘There are data that suggest….’

Attitudes/willingness towards digital?

**Other things we would want to know**

Demographics (gender, age, ethnicity), place of residence (rural/urban/IMD score – need postcode), psychosocial aspects, social support and friends/family, physical fitness, engagement with health behaviours, employment status, smoking, alcohol consumption.

**Other things to consider**

Questions will need to be adapted for carer participants where appropriate.

**Carer Interview Topic Guide**

**Introductory questions**

Please could you introduce yourself.

Tell me about the area where you live.

Tell me about the types of care you provide in general for your friend/family member with lung cancer? Prompts include emotional support, physical care, chores, travel etc.

**Questions on/experience of referral route**

When did you first become involved in caring for your friend/family member with lung cancer?

What was you experience as a carer when your friend/family member:

- First developed/notice symptoms?
- Was diagnosed?
- During referral for treatment following diagnosis?

What types of test were involved in the diagnosis process? X-ray, CT scan, PET-CT scan, Biopsy?

When and how were was your friend/family member diagnosed? What was their confirmed diagnosis?

When were they referred for treatment following their diagnosis?

Did they see a specialist within two weeks of a suspected lung cancer diagnosis?

How easy was it for them to get an appointment, how long did this take?

What types of support did you provide as a carer during these processes? (symptom development/diagnosis/referral for treatment)

Experiences on the conversation/communication around diagnosis/treatment with primary care/GP and specialist?

**Knowledge and willingness to adhere to necessary elements of a diagnostic and treatment pathway**

Can you tell me what you know about what options were made available to your friend/family member for their treatment?

- Chemotherapy, Radiotherapy, Surgery, Targeted Therapy, Immunotherapy

How much did you understand about these options? How much do you think your friend/family member understood about these options?

What aspects of treatment were explained to you? (prompts: side effects, effectiveness) Who were they explained by? (Friend/family member, consultant, nurse)

How involved did you feel in the discussions about your friend/family member’s options and the final treatment decision? How involved did you want to be? What did you feel your role as a friend/family member was during this time?

What did your friend/family member want to do with regards to their treatment? Did this differ from your view? Did this differ from their treatment plan? How did your friend/family member make a final decision about their treatment?

- Time/frequency of treatment
- Perceptions of treatment efficacy
- Possible side effects and tolerability
- Effects of treatment on family/social life
- Impact on work/finances/caring responsibilities
- Health status at the time (e.g., comorbidities, lung cancer symptoms)
- Opinion/recommendation of clinician
- Experiences of others/existing perceptions of treatment
- Views/influence of family/friends.

To what extent did you feel your friend/family member relied on you to help make a decision?

Who did your friend/family member speak to/consult about their diagnosis/treatment? Who did you speak to/consult about your friend/family member’s diagnosis/treatment?

Can you talk me through your experience of those conversations?

Were you aware of any support for your friend/family member? What types? Did they want to/were they able to access this support? Did you want them to access this support? In what ways were you involved in helping them to access this support?

Were you aware of any support for you as a person involved in caring for your friend/family member? What types? Did you want to/were you able to access this support?

Can you think of anything that could have been done better about your friend/family member’s diagnosis/treatment?

Can you think of anything that was done well about your friend/family member’s diagnosis/treatment and should be continued?

Can you talk me through your friend/family member’s experience of travelling to and from their diagnostic/treatment appointments? In what ways have you been involved in helping them with the travel? Have they faced any particular challenges? Have you faced any particular challenges?

- Ease/difficulty
- Talk me through how you made your journeys/experiences of them
- How far did you (would you have been prepared) to travel for your treatment?
- How did you feel about that?
- Did you have/want someone to go with you?
- Do you have access to a vehicle/public transport?
- Financial impact of treatment? (i.e., travel costs, lost work/other implications)
- Impact of health status/wellness/symptoms/comorbidities
- Views on time commitment
- Impact of competing responsibilities/commitments
- Managing different appointments at different places with different teams?
- Consistency and clarity (or not) of correspondence about appointments

Your friend/family member decided not to do [X], could you tell me about the reasons behind that decision? How do you feel about that decision as a friend/family member? Is there anything that could have been done differently?

Decision making – how location has influenced this?

Health status of you friend/family member – wellness/ability to travel

Your own health status – wellness/ability to travel

COM-B model – motivation –how motivated to you feel to be involved in helping your friend/family member through this diagnosis and treatment journey? What factors influence your motivation? Are there ever any challenges that stop you feeling motivated to be involved? How motivated do you feel your friend/family member is to go through their diagnosis and treatment journey? What factors do you think influence their motivation?

Perceptions of treatment efficacy and tolerability

Other people’s experiences – perhaps using third person to frame questions.

Towards end – suggestions, thinking about patients in the future, anything living in your area you would want? And thinking about other friends/family members supporting patients in your area – what kind of support would you want?

Smoking behaviour – Health professionals’ attitudes towards smoking?

‘Myths’

Using data/evidence to frame questions ‘There are data that suggest….’

Attitudes/willingness towards digital?

**Other things we would want to know**

Demographics (gender, age, ethnicity), place of residence (rural/urban/IMD score – need postcode), psychosocial aspects, social support and friends/family, physical fitness, engagement with health behaviours, employment status, smoking, alcohol consumption.
